# Supplementary material for: Effects of Coenzyme Q10 on the Biomarkers (Hydrogen, Methane, SCFA and TMA) and Composition of the Gut Microbiome in Rats
Source: Pharmaceuticals (Basel). 2023 May 2;16(5):686. doi: 10.3390/ph16050686 (PMC10223968; doi:10.3390/ph16050686)
Supplement: Supplementary file 1 [file pharmaceuticals-16-00686-s001.zip › pharmaceuticals-2325178-supplementary.pdf]

Figure S1. Rarefaction curves.

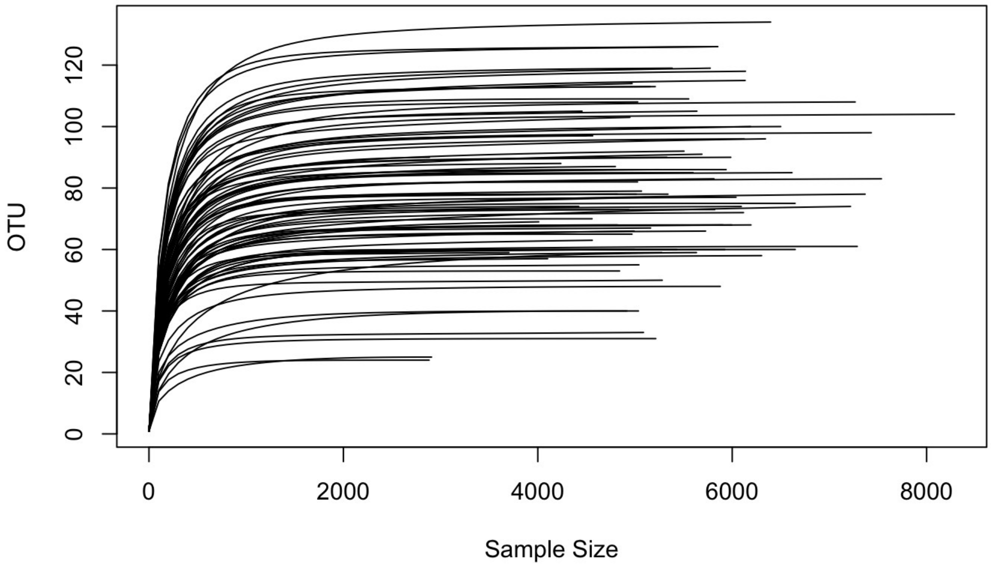

Table S1. Relative abundance of top phyla at pre- and post-treatment stages in all experimental groups.

	Control		Vehicle		CoQ10	
	**Pre**	**Post**	**Pre**	**Post**	**Pre**	**Post**
Firmicutes	65,1535	75,50571428571	73,6765	79,18025	66,1734375	68,9211875
Bacteroidota	27,37571428571	19,19233571428	17,00275	13,18883333333	20,2915625	22,8609375
Actinobacteriota	1,975428571429	1,248928571428	1,625166666666	1,26725	2,371625	1,6203125
Proteobacteria	0,673142857143	0,1665	0,45475	0,574583333333	0,5405	0,6205
Campilobacterota	0,823857142857	0,958071428571	1,881416666666	1,217166666666	2,4218125	0,6204375
Desulfobacterota	1,324928571428	0,723357142857	3,410666666666	1,455083333333	5,551	2,8890625
Spirochaetota	2,291642857142	1,945428571428	1,373916666666	2,838583333333	2,0198	1,8905
Unclassified taxa	0,381785714285	0,259664285714	0,574833333333	0,278249999999	0,41499999	0,577062500000

Table S2. Analysis of differential abundance at genera level.

	control			vehicle			coenzyme Q10		
Taxon	*ancombc*	*deseq2*	*wilcoxon*	*ancombc*	*deseq2*	*wilcoxon*	*ancombc*	*deseq2*	*wilcoxon*
[Eubacterium] hallii group	+							+*	
[Eubacterium] siraeum group	+								
[Ruminococcus] torques group							+		
Alistipes						+*	+		
Allobaculum					+*	+*			
Anaerobiospirillum	+								
Anaerovibrio								+*	
Bifidobacterium	+	+*	+*					+*	
Blautia						+*			
Butyricicoccus						+*			
Campylobacter					+*				
Catenisphaera	+								
Chlamydia						+*			
Christensenellaceae R-7 group		+*			+*				
Clostridia UCG-014					+*				
Clostridium sensu stricto 1		+*						+*	
Colidextribacter	+		+*						
Coprococcus	+					+*			
Desulfovibrio								+*	
Dorea							+	+*	
Dubosiella							+		
Enterorhabdus				+	+*		+		
Erysipelatoclostridium	+	+*	+*		+*		+		+*
Erysipelotrichaceae UCG-006				+					
Eubacterium.xylanophilum.group						+*			
Faecalibacterium								+	
Faecalibaculum	+								
GWE2-31-10	+						+		
Helicobacter							+	+*	+*
Incertae Sedis	+								
Lachnospiraceae AC2044 group	+		+*	+			+		+*
Lachnospiraceae NK3A20 group				+					
Lachnospiraceae ND3007 group						+*			
Lawsonia	+			+			+	+*	
Methanobrevibacter				+	+*		+	+*	+*
Mogibacterium				+					
Monoglobus				+		+*			+*
Mucispirillum				+		+*			+*
Mycoplasma	+								
Negativibacillus				+					
Olsenella						+*			
Oscillibacter	+			+			+		
Oscillospira	+					+*	+		
Parasutterella	+			+					
Peptoclostridium	+			+		+*		+	
Peptococcus	+			+	+	+*	+		
Prevotellaceae UCG-001 group							+		
Prevotellaceae Ga6A1 group						+*			
Pygmaiobacter				+			+	+*	+*
Rikenellaceae RC9 gut group								+*	
Roseburia							+		
Ruminococcus							+	+*	
Senegalimassilia	+			+	+*		+		
Shuttleworthia	+						+		
Solobacterium			+*				+		
Staphylococcus				+					
Streptococcus						+*			
Subdoligranulum								+*	
Sutterella	+								
Turicibacter	+	+*		+				+*	+*
Erysipelatoclostridiaceae UCG-004 group						+*			
Oscillospiraceae UCG-005 group		+*			+*	+*			
Ureaplasma				+					+*

'+' means that adjusted p-value for the chosen method is < 0.05; '+*' means that unadjusted p-value for the method is < 0.05, while adjusted is > 0.05; blank cells - both adjusted and unadjusted p-values > 0.05; taxa for which at least to algorythms showed differential abundance before and after treatment ara highlighted with pale green
